# A Survey Exploring Reasons behind Immunization Refusal among the Parents and Caregivers of Children under Two Years Living in Urban Slums of Karachi, Pakistan

**DOI:** 10.3390/ijerph191811631

**Published:** 2022-09-15

**Authors:** Asif Khaliq, Alfaraz Ashraf Elahi, Asima Zahid, Zohra S. Lassi

**Affiliations:** 1 Department of Health & Hospital Management, Institute of Business Management, Karachi 75190, Pakistan; 2 School of Public Health & Social Work, Queensland University of Technology, Brisbane, QLD 4059, Australia; 3 Robinson Research Institute, The University of Adelaide, Adelaide, SA 5005, Australia

**Keywords:** immunization, refusal, reasons, parents, caregivers, urban slums, children, Karachi

## Abstract

This study assesses the experiences of parents/caregivers regarding the refusal to childhood immunization. A cross-sectional study was conducted among the parents/caregivers of children under two years old from January 2019 to June 2019 who were residents of either Pathan Colony or Orangi Town, Karachi. In this study, the data collectors targeted parents/caregivers of 440 households who showed a refusal mark “R” in the Expanded Program of Immunization (EPI) H-chalking system. These households were approached using a 30 × 7 multistage-stratified-cluster random sampling technique and were interviewed using a structured questionnaire. The study sample produced two different types of refusals: true refusal (absence) and potential refusal (presence), based on the absence and presence of a vaccination card at the time of the survey. Multivariate logistic regression was used to analyze the data using Jamovi (V-1.6.13). A total of 230 households consented to participate in this study, of which 141 (61.3%) represented true refusals, while 89 (38.7%) represented potential refusals. More than half of the participants from both groups complained about fever and pain at the injection site following immunization. The use of alternative medicines and a history of adverse events following immunization (AEFI) were associated with increasing the odds of immunization refusals by four-to-five fold. However, advanced paternal age, a long distance to the clinic, a lack of trust in government, and the influence of community/religious leaders were associated with lower immunization refusal odds. Thus, an unawareness about self-limiting vaccine-related adverse events, the use of alternative medicines, and an increased concern about the safety and efficacy of vaccines were found to be barriers to immunization, which can be improved by increasing public awareness through media campaigns and policy reform.

## 1. Introduction

Immunization is an effective tool for preventing and controlling morbidity and mortality associated with vaccine-preventable diseases (VPDs) [1]. Globally, immunization has prevented more than two million deaths among children under five years of age [2,3]. Besides health benefits, immunization has many economic benefits, such as reducing illness costs, shortening the length of stay, and improving the quality of an adjusted life year [4,5]. Despite multiple benefits, the therapeutic potential of immunization against various VPDs is questionable to various communities, leading to caregivers/parents’ refusal of vaccine administration [6].

Immunization refusal is a global threat, defined as a deliberate delay or refusal of vaccination by parents and/or caregivers and/or the community without any vaccine logistics or supply issues [7,8]. Factors such as illiteracy, fear of adverse effects, and religious and cultural beliefs are mainly associated with immunization refusal [9,10,11,12]. Similarly, negative rumors (vaccines cause infertility/impotency), lack of trust regarding vaccine efficacy, and concerns regarding adverse events following immunization (AEFI) also contribute to immunization refusals [12,13,14]. The problems related to immunization refusal in an individual and a community can be averted by improving the immunization coverage to ≥80%, and an improvement in the immunization coverage has the potential to prevent around 15 million deaths among children below five years of age [2,3,8,15].

In 1988, the World Health Organization (WHO), in consultation with the United States Centre for Disease Control and Prevention (US-CDC), United Nations Children Funds (UNICEF), Rotary International, and various other national and local bodies, introduced the concept of supplemental immunization activities (SIAs) to improve the immunization coverage at a regional, national, and global level [16]. SIA implementation was the cornerstone for improving immunization coverage, reducing disease transmission, and uplifting immunity in most countries in the world [16,17]. However, certain countries, including Pakistan, failed to show promising outcomes of SIA implementation [17]. Compared to routine immunization activities, Pakistan has historically reported a higher number of immunization refusal incidents during mass immunization campaigns and SIAs [13,16,18]. For this reason, Pakistan is still endemic to certain VPDs, such as polio and measles, while these VPDs have been eradicated globally [16,19,20]. Therefore, this study aims to examine the factors that influence the decisions of parents/caregivers of children aged below two years regarding vaccination during SIAs.

## 2. Methodology

### 2.1. Study Design, Duration, Setting, and Population

**Figure 1:**
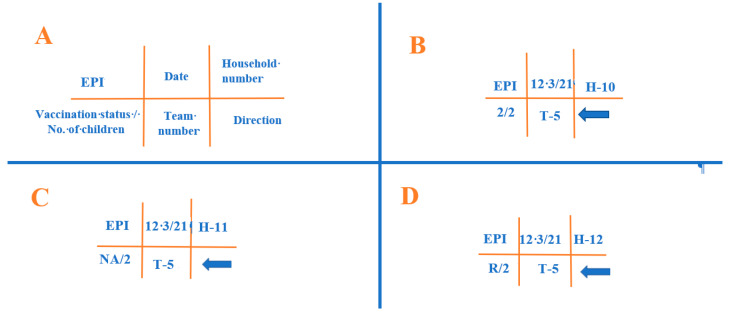
Description of H-household chalking used by the mobile vaccination team across Pakistan. Figure 1 explains different sections of the H-chalking developed by the Expended Program of Immunization to assess mobile vaccination activity. (**A**) shows six sections of H-chalking. The top three sections show the EPI, date of visit of vaccination team, household number; meanwhile, the bottom section shows vaccination status of children in the household visited, vaccination team number, and direction of movement of vaccination team. (**B**–**D**) describes the example of H-chalking in three different households. It can be interpreted that EPI team 5 visited household 10, 11, and 12 on 12 March 2021, and all these households have two children according to Town health registry. Children in H-10 received vaccines (**B**), children were not present at time of team visit in H-11 (**C**), and the parents/caregivers refused to vaccinate their children to the vaccination team in H-12 (**D**).

A community-based door-to-door household survey was performed among parents and caregivers of children under two years of age who were residents of either Pathan Colony or Orangi Town, Karachi, from January 2019 to June 2019 to investigate the reasons behind the vaccine refusals for their children. In this study, the research team captured houses marked with an “R” on the H-household chalking used by the Expanded Program of Immunization (EPI) teams. The EPI team of Pakistan designed an H-household chalking system, which helps to identify vaccination activities performed by each vaccination team in the designated household. Thus, H-chalking can identify the EPI team, the number of children, and the refusals (defaulters) from each household. The H-household chalking has six different sections, and the description of H-household chalking is presented in Figure 1.

The “R” sign in the H-household chalking system indicates “Refusals”. The study team identified two different types of refusals, based on the presence or absence of a vaccination card. The availability of vaccination was considered to be indicator in the classification of true refusals and potential refusals, because all the public and private hospitals, clinics, and community centers of Pakistan issued a vaccination card to the parents/caregivers of all vaccinated children for their immunization record and follow-up visits. Thus, based on the presence or absence of a vaccination card, the research team identified two different types of refusals: true refusal and potential refusal. ***True refusal:*** The research team classified all the households who did not show vaccination cards at the time of visit as true refusals, which means they were not given a vaccination card because they did not bring their children for routine immunization. ***Potential refusal:*** The refusals who refused child vaccination from the mobile vaccination team during the SIAs instead provided vaccination to their children from vaccination centers. Moreover, the potential refusers showed vaccination cards to the research team. Households with a language barrier to Urdu were excluded from this study’s survey.

### 2.2. Sample Size and Sampling Methods

Since no official report exists regarding the immunization refusal rate, the national immunization coverage was considered for calculating the sample size of this study. Considering the national immunization coverage of 84% [21], confidence interval (CI) of 95%, and margin of error of 5%, the sample size calculated was 224 [22,23]. The research team extrapolated the estimated sample size to double because of participants’ ineligibility and consent refusals. Thus, a total of 440 households were approached to selecting the participants of this study.

A 30X7 multistage-stratified-cluster random sampling technique was used to assess the precision of an immunization program. The research team initially created two strata to represent each study area, i.e., Orangi Town and Pathan Colony. However, to select the target population of this study, the data collector with the aid of different community stakeholders, such as union council members and the mobile vaccination team, received a list of households. Based on the list of households, the research team then created 30 clusters from each area, and these clusters were composed of around 150–250 households. From each cluster, the data collector selected the targeted household of this study by reading the H-chalking identified by the EPI team of Orangi Town and Pathan Colony. For systematic selection, every seventh household with a refusal mark “R” in the H-chalking system were selected for the data collector. The H-chalking was placed either outside the entrance door or on the wall of each household. After identifying targeted households with a refusal mark “R”, the data collector presented verbal consent to determine the households’ eligibility, and to each eligible household, the data collector issued a written consent form for study participation.

### 2.3. Data Collection Instrument and Procedure

An objective-driven self-made questionnaire was designed after an extensive literature review to assess the vaccination refusals among the parents or caregivers of children in selected areas of Karachi. The questionnaire had different sections, of which the first three sections described the participant screening, eligibility assessment, and enrolment. A four-digit enrolment number was assigned to each parent/caregiver who consented to participate in the study.

From each participant enrolled, information collected was related to the socio-demographic profile, parents’ experiences, and behaviors associated with the immunization refusals. Content related to the parents’ behavior regarding immunization was adapted from the study by Khaliq et al. in 2016 [24]. In addition to the survey questions, a separate section for data quality control and quality assurance was added to identify the person who filled, reviewed, and entered the form into the data entry software.

The questionnaire was then translated Into Urdu—the local language. A pediatric public health specialist checked the content validity of the questionnaire, and further validation was conducted by the Management Review Committee (MRC) of the Institute of Business Management (IoBM), Pakistan. The face validity and reliability of the questionnaire were tested on 10% of the sample (pilot data were not included in the final analysis). The Cronbach alpha value after pilot testing for 20 items was 0.714 by the test-re-test method, indicating good reliability [25].

### 2.4. Data Analysis

The data were first analyzed descriptively. The outcome variable of this study was vaccine refusal: true refusal and potential refusal. Unadjusted and adjusted odds ratios (oRs) were calculated using binomial logistic regression, and all the statistically insignificant variables with a *p*-value over 0.05 were removed sequentially from the model using the backward elimination method. In the final model, only significant predictor variables were considered. The data were analyzed using Jamovi software version 1.6.13 (www.jamovi.org) [26].

### 2.5. Legal and Ethical Consideration

Ethical clearance was sought from the MRC of the MBA Health and Hospital Management (MHM) program at IoBM, Karachi, Pakistan (21905-2019-MBA-MHM).

## 3. Results

### 3.1. Characteristics of Study Population

**Figure 2:**
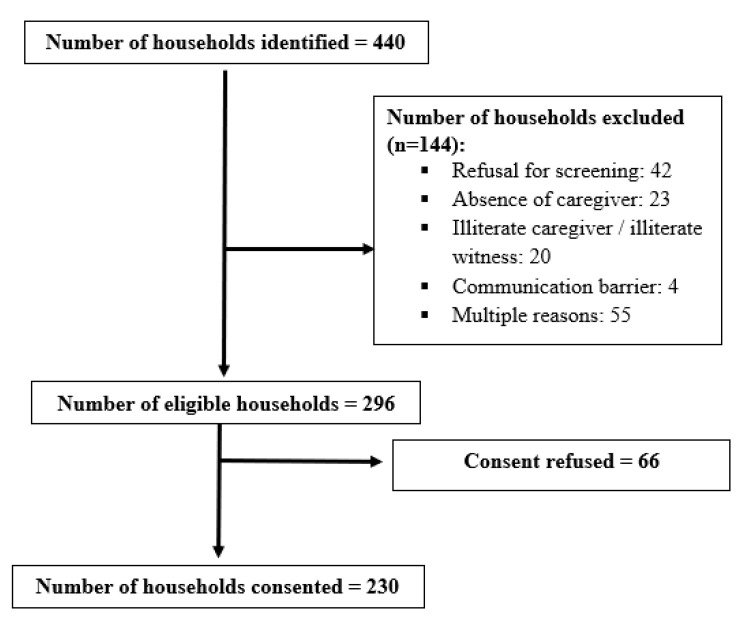
Participants tracking and eligibility screening.

A total of 440 households with a refusal mark “R” were identified; 67.2% (n = 296) were eligible. Of the eligible households, 230 consented to participate (53%) (Figure 2).

Of the 230 parents/caregivers interviewed, there were 141 (61.3%) true refusals and 89 (38.7%) potential refusals. The mean number of children who refused vaccination was 2.77 + 1.89 years, and the majority (64.8%) belonged to low socioeconomic backgrounds. The mean age of their mother and father was 30.4 ± 6.26 years and 37.9 ± 7.47 years, respectively. Most mothers were not working (97.8%) and were not educated (81.3%). Meanwhile, 97.4% of the fathers were working and 63% of them had received some education.

Around 60% of both true and potential refusals reported having a fever and pain at the injection site as common AEFI, while inflammation, allergic reactions, hospitalization, disability, and death were reported mainly by true refusals (Table 1).

The reasons for not vaccinating among true refusals were the cost, side effects and contraindications of vaccines, rumors, and alternative medicines. However, the distance to clinics, reduced clinic timing, community/religious leader influence, and a lack of trust in the government were mainly reported by potential refusals. Both true and potential refusals reported that new vaccines are not safe for the health of their children (Table 1).

### 3.2. Assessing the Association of Immunization Refusals with Parent’s Experiences

After adjusting for confounders, advanced paternal age, long clinic distance, lack of trust in the government, and influence of community/religious leaders were significantly associated with reduced odds of immunization refusal (*p* < 0.05). In contrast, the use of alternative medicines and a history of vaccine-related side effects, allergic reactions, and disability were significantly associated with increased odds of immunization refusals (*p* < 0.005) (Table 2).

## 4. Discussion

This study has identified the reasons for immunization refusal among the parents/caregivers of children under two years of age. The findings reveal that parents/caregivers refused to vaccinate their children because of a history of AEFI, vaccine-related side effects (VREs), and complementary and alternative medicine (CAM) adoption to prevent VPDs. Earlier studies also support the notion ed that a lack of confidence in vaccine preventive efficacy and a fear of VREs majorly contribute to immunization refusal [27,28,29].

Earlier studies have reported a significant association between maternal education, empowerment, and wealth status with immunization practices [30,31,32]. Studies from Pakistan have reported that an increase in maternal education, empowerment, and wealth index significantly reduces immunization refusals [33,34]. Similarly, a study conducted in Italy showed an association of immunization refusal with poor education levels and poor socioeconomic status [35]. A systematic review by Forshaw, et al. (2017) further strengthens the relationship of immunization refusal with maternal education by indicating a protective role of maternal education in immunization adherence and immunization coverage [36]. However, this study reported a non-significant relationship between maternal factors and immunization practices: it is important to highlight because, in Pakistan, women are not empowered to make decisions, and a multitude of socio-cultural factors, such as male dominancy, women illiteracy, and lack of family support, tend to amplify the dependency of women on family members [37,38]. Sebahat and Nadi (2006) presented a lack of maternal empowerment as a prime reason for immunization refusal [30]. Thus, empowerment of women is fundamental for socio-cultural reform [37]; thus, there is a need to empower women through social advocacy and cultural reforms.

This study identified young paternal age as an important predictor of immunization refusal. A systematic review conducted by Sadaf et al. (2013) reflected that immunization refusal is continuously emerging worldwide among parents [39]. It is evident from past research that parents have refused to immunize their children due to a fear of VREs, a lack of trust in government, and religious skepticism, all of which majorly contribute to immunization refusal [40,41]. This study reported a promotive role of religious and political leaders and the government in effectively implementing the immunization program. Conversely, many studies conducted in Pakistan and other regions of the world reported a lack of confidence and trust in the government as one of the prime reasons for immunization refusal [27,42]. However, in their study, Durenaz et al. (2020) also stated that religion is not a prime factor for immunization refusal; instead, parents’ concerns regarding vaccine safety and efficacy plays a significant role [28]. Similarly, a qualitative study by Keshet and Popper-Giveon revealed that religious scholars advocate immunization, but parents/caregivers refuse to vaccinate their children due to their orthodox thinking [43]. This depicts the need to educate orthodox parents/caregivers about the health benefits of immunization; thus, health education will then expand immunization adherence and immunization acceptance among the refusals.

The distance to vaccination centers is an important determinant for immunization practices. This study identified that an increase in the distance to the vaccination center significantly decreased the odds of immunization refusal, which signifies that parents appreciate the door-to-door vaccination service. Earlier studies from Pakistan have also reported that a long distance to the vaccination center is a barrier to immunization [28,29].

An insignificant relationship between immunization refusal with commonly reported AEFI, such as fever, swelling, pain at the injection site, diarrhea, and cold/cough, was reported. This reflects the fact that commonly reported AEFI are not associated with immunization refusals. However, a significant relationship was reported between immunization refusal with hospitalization and paralysis (disabilities). Although, no association between death following immunization has been reported in a systematic review [44]. Similarly, a vaccine trial conducted in Pakistan showed no cases of hospitalization, disabilities, or death following immunization among children [45]. Another study from Zambia revealed that the history of vaccine-related side effects is a barrier to immunization success [46]. Moreover, it was also identified in this study that parents prefer CAM and refuse the vaccination because they believe CAM is the safest method for preventing and managing illness [47]. Thus, a history of severe and uncommon vaccine-related side effects and the use of CAM contributes to immunization refusals by parents. In this regard, it is essential to build the trust of parents/caregivers regarding vaccine benefits, vaccine side effects, and vaccination schedules by providing vaccine-related education and information materials.

### Study Limitations and Future Directions

Immunization refusal has received global attention as vaccines help prevent disease and save millions of people worldwide. The risks and benefits are the most cited reasons behind hesitancy internationally [48]. Although the results of this study provide an insight into vaccine refusal and the reasoning among those who refuse to vaccinate their infants for routine pediatric immunization, there were certain limitations that we feel may have weakened the internal validity of this study, i.e., the cross-sectional study design and all the information provided by parents or other caregivers was only considered for data collection. Moreover, the data collection method for certain variables was not appropriate. For example, there was no cut-off to the distance from the clinic, the timing of the clinic, and the waiting time in the clinic. If these variables had cut-offs, it could have helped us propose recommendations.

Moreover, parents’ education was assessed subjectively and due to this, no significant turn points for future studies can be made from the study’s maternal and paternal education findings. This study has represented the views of parents living in Pathan Colony and Orangi Town, Karachi, Pakistan, and conclusions from two localities of Karachi cannot be generalized to the whole population of the city. Additionally, opinions from key stakeholders, such as community workers, community physicians, union council members, and religious leaders, were not considered. Therefore, there is a need for more studies that assess immunization refusal during the routine and the SIAs, and this will help the exploration of the ground realities of immunization refusal from a specific campaign.

## 5. Conclusions

Immunization refusal is a basic reason for low immunization coverage and the emergence of diseases that can be prevented through immunization. Poverty and illiteracy act as major barriers to vaccination. Unawareness about self-limiting vaccine-related adverse events, i.e., fever, pain and swelling at the injection site, a lack of trust in the government, and an increased concern about the safety and efficacy of vaccines were found to be barriers to routine immunization. Thus, parents refuse to vaccinate their children due to many factors.

## Figures and Tables

**Table 1:** Characteristics of parents/caregivers of children who refused vaccination (n = 230).

Variables	Categories	Total (n = 230)	Potential Refusal (n = 89)	True Refusal (n = 141)
**Maternal factors**				
**Maternal age**		30.4 ± 6.26	31.3 ± 6.39	29.9 ± 6.13
**Mother education**	Educated	43 (18.7)	15 (16.8)	28 (19.8)
	Not educated	187 (81.3)	74 (83.1)	113 (80.2)
**Mother employment**	Working	5 (2.2)	86 (96.6)	139 (98.5)
	Not working	225 (97.8)	3 (3.4)	2 (1.5)
**Paternal factors**				
**Paternal age**		37.9 ± 7.47	40.1 ± 8.46	36.6 ± 6.43
**Father education**	Educated	145 (63)	58 (65.1)	87 (61.8)
	Not educated	85 (37)	31 (34.9)	54 (38.2)
**Father employment**	Working	224 (97.4)	87 (97.7)	137 (97)
	Not working	6 (2.6)	2 (2.3)	4 (3)
**Household factors**				
**Under two children**		2.77 ± 1.89	2.96 ± 1.89	2.65 ± 1.89
**Socioeconomic status**	Low	149 (64.8)	58 (65.1)	91 (64.5)
	Middle	78 (33.9)	30 (33.7)	48 (34)
	High	3 (1.3)	1 (0.1)	2 (1.5)
**AEFI Experienced**				
**Fever**	Yes	149 (64.8)	54 (60.6)	95 (67.3)
	No	81 (35.2)	35 (39.4)	46 (36.7)
**Inflammation**	Yes	136 (59.1)	46 (51.6)	90 (63.8)
	No	94 (40.9)	43 (48.4)	51 (46.2)
**Pain at the injection site**	Yes	132 (57.4)	50 (56.1)	82 (58.1)
	No	98 (42.6)	39 (45.9)	59 (41.9)
**Self-limiting illnesses ^∞^**	Yes	136 (59.1)	54 (60.6)	82 (58.1)
	No	94 (40.9)	35 (39.4)	59 (41.9)
**Allergic reactions ^¥^**	Yes	50 (21.7)	12 (12.5)	38 (26.9)
	No	180 (78.3)	77 (86.5)	103 (73.1)
**Hospitalization**	Yes	52 (22.6)	11 (12.4)	41 (29.1)
	No	178 (77.4)	78 (87.6)	100 (70.9)
**Disability**	Yes	29 (12.6)	5 (5.7)	24 (17)
	No	201 (87.4)	84 (94.3)	117 (83)
**Death**	Yes	18 (7.8)	6 (6.8)	12 (8.5)
	No	212 (92.2)	83 (93.2)	129 (91.5)
**Reasons for refusal**				
**Long distance to the clinic**	Yes	91 (39.6)	40 (44.9)	51 (36.2)
	No	139 (60.4)	49 (56.1)	90 (63.8)
**Reduced clinic timing**	Yes	74 (32.2)	34 (38.2)	40 (28.4)
	No	156 (67.8)	55 (61.8)	101 (71.6)
**The high cost of the vaccine**	Yes	79 (34.3)	24 (26.9)	55 (39)
	No	151 (65.7)	65 (73.1)	86 (61)
**Rumors about vaccine use**	Yes	180 (78.3)	60 (67.4)	120 (85.1)
	No	50 (21.7)	29 (22.6)	21 (14.9)
**Influence of religious or community leaders on not vaccinating**	Yes	162 (70.4)	70 (78.6)	92 (63.3)
	No	68 (29.6)	19 (21.4)	49 (34.7)
**Lack of trust in government**	Yes	35 (15.2)	27 (31.4)	8 (5.7)
	No	195 (84.8)	62 (69.6)	133 (94.3)
**New vaccines are not safe**	Yes	133 (57.8)	52 (58.4)	81 (57.5)
	No	97 (42.2)	37 (41.6)	60 (42.5)
**Vaccine contraindication**	Yes	177 (77)	64 (71.9)	113 (80.2)
	No	53 (23)	25 (28.1)	28 (19.8)
**The vaccine caused adverse events**	Yes	177 (77)	63 (70.7)	114 (80.9)
	No	53 (23)	26 (29.3)	27 (19.1)
**An alternative way for disease prevention**	Yes	60 (26.1)	10 (11.2)	50 (35.5)
	No	170 (73.9)	79 (88.8)	91 (64.5)

^∞^ = Diseases such as diarrhea, cold, flu, irritability (crying), and malaise were considered self-limiting illnesses, ^¥^ = fast breathing, cough, wheezing, rashes, and swelling of face and eyes.

**Table 2:** Experiences associated with true immunization refusal.

Variables	Categories	Unadjusted OR (95% CI)	Adjusted OR (95% CI)
**Maternal factors**			
**Mother age**	-	0.96 (0.92 to 1.01)	-
**Mother education**	Yes	Ref	-
	No	0.81 (0.41 to 1.63)	
**Mother employment**	Yes	2.42 (0.39 to 14.80)	-
	No	Ref	
**Paternal factors**			
**Father age**	-	0.93 (0.90 to 0.97) *	0.93 (0.89 to 0.97) *
**Father education**	Yes	Ref	-
	No	1.16 (0.66 to 2.02)	
**Father employment**	Yes	Ref	-
	No	1.27 (0.22 to 7.08)	
**Household factors**			
**Under 5-year children**		0.91 (0.79 to 1.06)	-
**Socioeconomic status**	Low	0.78 (0.06 to 8.85)	-
	Middle	0.80 (0.06 to 9.21)	
	High	Ref	
**AEFI Experienced**			
**Fever**	Yes	1.34 (0.77 to 2.33)	-
	No	Ref	
**Inflammation**	Yes	0.91 (0.54 to 1.55)	-
	No	Ref	
**Pain at the injection site**	Yes	1.08 (0.63 to 1.85)	-
	No	Ref	
**Self-limiting illnesses ^∞^**	Yes	1.65 (0.96 to 2.83)	-
	No	Ref	
**Allergic reactions ^¥^**	Yes	2.37 (1.16 to 4.83) *	4.01 (1.54 to 10.41) *
	No	Ref	Ref
**Hospitalization**	Yes	2.91 (1.40 to 6.02) *	-
	No	Ref	
**Disability**	Yes	3.45(1.26 to 9.40) *	4.66 (1.42 to 15.28) *
	No	Ref	Ref
**Death**	Yes	1.30 (0.46 to 3.59)	-
	No	Ref	
**Reasons for non-vaccination**			
**Long distance to the clinic**	No	0.69 (0.40 to 1.19)	0.41 (0.19 to 0.84) *
	Yes	Ref	Ref
**Reduced clinic timing**	Yes	0.64 (0.36 to 1.12)	-
	No	Ref	
**The high cost of the vaccine**	Yes	1.73 (0.97 to 3.09)	-
	No	Ref	
**Rumors about vaccine use**	Yes	2.76 (1.45 to 5.25) *	-
	No	Ref	
**Influence of religious or community leaders**	Yes	0.51 (0.27 to 0.94) *	0.36 (0.16 to 0.82) *
	No	Ref	Ref
**Lack of trust in government**	Yes	0.13 (0.05 to 0.32) *	0.11 (0.03 to 0.32) *
	No	Ref	Ref
**New vaccines are not safe**	Yes	0.96 (0.56 to 1.64)	-
	No	Ref	
**Vaccine contraindication**	Yes	1.58 (0.84 to 2.93)	-
	No	Ref	
**The vaccine caused adverse events**	Yes	1.74 (0.93 to 3.24)	2.48 (1.11 to 5.52) *
	No	Ref	Ref
**The alternative way of disease prevention**	Yes	4.34 (2.06 to 9.12) *	4.63 (1.87 to 11.42) *
	No	Ref	Ref

^∞^ = diseases, such as diarrhea, cold, flu, irritability (crying), and malaise were considered self-limiting illnesses, ^¥^ = urticaria, rashes, shortness of breath within 30 to 60 min following vaccination * = significant association having *p*-value < 0.05.

## Data Availability

The data of this study is not publicly available, but can be accessed on request from the corresponding author.
